# Glucagon-like peptide-1 attenuates diabetes-associated osteoporosis in ZDF rat, possibly through the RAGE pathway

**DOI:** 10.1186/s12891-022-05396-5

**Published:** 2022-05-17

**Authors:** Yanzhen Cheng, Peng Liu, Qianru Xiang, Jiamin Liang, Huafeng Chen, Hua Zhang, Li Yang

**Affiliations:** 1 Department of Endocrinology and Metabolism, Zhujiang Hospital, the Second School of Clinical Medicine, Southern Medical University, Guangzhou, 510000 Guangdong People’s Republic of China; 2 Department of Cardiology, Zhujiang Hospital, the Second School of Clinical Medicine, Southern Medical University, Guangzhou, 510000 Guangdong People’s Republic of China; 3 Department of Nutrition, Zhujiang Hospital, the Second School of Clinical Medicine, Southern Medical University, Guangzhou, 510000 Guangdong People’s Republic of China

**Keywords:** Glucagon-like peptide-1, Advanced glycation endproducts, Diabetes, Osteoporosis, Liraglutide

## Abstract

**Background:**

Diabetes-associated osteoporosis are partly caused by accumulation of advanced glycation endproducts (AGEs). Glucagon-like peptide-1 (GLP-1) has been shown to regulate bone turnover. Here we explore whether GLP-1 receptor agonist (GLP1RA) can have a beneficial effect on bone in diabetes by ameliorating AGEs.

**Methods:**

In the present study, we evaluated the effects of the GLP-1 receptor agonist liraglutide, insulin and dipeptidyl peptidase-4 inhibitor saxagliptin on Zucker diabetic fatty rats. Meanwhile, we observed the effect of GLP-1 on AGEs-mediated osteoblast proliferation and differentiation and the signal pathway.

**Results:**

Liraglutide prevented the deterioration of trabecular microarchitecture and enhanced bone strength. Moreover, it increased serum *Alpl*, *Ocn* and *P1NP* levels and decreased serum *CTX*. In vitro we confirmed that GLP-1 could attenuate AGEs-mediated damage in osteogenic proliferation and differentiation. Besides, GLP-1 down-regulated the ROS that caused by AGEs and the mRNA and protein expression of *Rage* .

**Conclusions:**

Altogether, our findings suggest that GLP-1 receptor agonist promotes osteoblastogenesis and suppresses bone resorption on obese type 2 diabetic rats to a certain degree. The mechanism of these effects may be partly mediated by AGEs-RAGE-ROS pathway via the interaction with GLP-1 receptor.

**Supplementary Information:**

The online version contains supplementary material available at 10.1186/s12891-022-05396-5.

## Background

Osteoporosis is a skeletal disorder characterized by decreased bone mass and damaged bone tissue microstructure, culminating in fragility fractures, pain and disability [1]. With the increasing incidence of diabetes, diabetes-associated osteoporosis has become more prevalent with high morbidity and fracture risks.

Glucagon-like peptide-1 (GLP-1) can be degraded by dipeptidyl peptidase-4 (DPP-4) and stimulates glucose-dependent insulin secretion [2]. Studies have shown that both DPP4 inhibitors and GLP-1 agonists have beneficial effects on the bone in patients [3, 4]. But the mechanisms remain elusive.

The formation and accumulation of advanced glycation end products (AGEs) were demonstrated as the main causes of diabetes-associated osteoporosis [5]. It’s reported that GLP-1 could attenuate the generation of AGEs-induced reactive oxygen species (ROS) [6–8]. It remains unknown whether GLP-1 can ameliorate the detrimental effects of AGEs in diabetes-related osteoporosis. Therefore, we aimed to identify the potential protective pathways that GLP-1 triggered to counteract AGEs-mediated damages both in vitro and in vivo.

## Methods

### Materials

Human GLP-1 _(7–36)_ (GLP-1), Exendin_(9–39)_ (exendin), the amebocyte lysate assay kit, dexamethasone, ascorbic acid, β-glycerophosphate, alizarin red, p-nitrophenyl phosphate,2′,7′-dichlorodihydrofluorescein diacetate (DCFH-DA), and 3-(4,5-dimethylthiazozyl)-2,5-diphenyl tetrazolium bromide (MTT) were purchased from Sigma (St. Louis, MO, USA). Rabbit anti-*Glp1r*, rabbit anti- *Rage*, mouse anti-*Gapdh*, rabbit anti-*Gapdh*, rabbit anti-Cyclophilin B, goat HRP-conjugated, rabbit HRP-conjugated, and Goat Anti-Rabbit IgG HRP 647 antibodies (Abcam, Cambridge, UK) were used, which were listed in Table 1. The protein marker (26634) was from ThermoFisher (California, USA). The BCA assay kit was from CoWin Biotech (Beijing, China); Dimethyl sulfoxide (DMSO) and TRIZOL reagent were obtained from Invitrogen (Buenos Aires, Argentina); Fetal bovine serum (FBS) and Dulbecco’s modified Eagle’s medium (DMEM) were all from HyClone (Logan, UT, USA); The PrimeScript® one-step Real Time Polymerase Chain Reaction (RT-PCR) kit and SYBR^®^ Premix Ex Taq™ II were from Takara Biotechnology (Dalian, China); RIPA lysis buffer was from Beyotime Biotech (Shanghai, China); the Detoxi-Gel column was from Pierce (Rockford, IL, USA); the ECL kit was from Applygen Technologies Inc. (Beijing, China). All other chemicals and reagents were purchased commercially and were of analytical grade.

**Table 1:** Antibodies list

Antibody Name	Dilution ratio	Product No.	Company
Rabbit anti-*Glp1r* antibody (rabbit monoclonal to *Glp1r*)	1:500 (WB) 1:200 (IF)	ab218532	Abcam Plc
Rabbit anti- *Rage* antibody (rabbit monoclonal to *Rage*)	1:500 (WB) 1:200 (IF)	ab216329	Abcam Plc
Mouse anti-*Gapdh* antibody (mouse monoclonal to *Gapdh*)	1:1000 (WB)	ab8245	Abcam Plc
Rabbit anti-*Gapdh* antibody (rabbit monoclonal to *Gapdh*)	1:1000 (WB)	ab181602	Abcam Plc
Rabbit anti-*Cyclophilin B* antibody (rabbit polyclonal to *Cyclophilin B*)	1:1000 (WB)	ab16045	Abcam Plc
Goat HRP-conjugated antibody (Goat Anti-Rabbit IgG HRP)	1:1000 (WB)	ab6721	Abcam Plc
Rabbit HRP-conjugated antibody (Rabbit Anti-Mouse IgG HRP)	1:1000 (WB)	ab6728	Abcam Plc
Goat Anti-Rabbit IgG HRP (Alexa Fluor® 647) preadsorbed	1:300 (IF)	ab150083	Abcam Plc

*WB* Western Blot, *IF* Immunofluorescence, *HRP* Horse radish peroxidase

### Animal experiments

All experiments and animal care procedures were carried out in accordance with the principles of laboratory animal care and were approved by the Animal Care and Use Committee of the Southern Medical University (Guangzhou, China). Twenty-eight male Zucker diabetic fatty rats [ZDF (fa/fa)] and 7 male Zucker lean control rats [ZLC (fa/+)] were purchased from the Laboratory Animal Center of Vital River [Beijing, China; license number, SYXK (Yue) 2011 0074] at 8 weeks of age. ZDF rats had ad libitum access to Purina LabDiet 5008 rat chow (protein 23%, fat 6.5%, carbohydrates 58.5%, fiber 4% and ash 8%) and purified water throughout the experiment. Rats at the age of 11 weeks with random blood glucose levels ≥300 mg/dl (16.7 mmol/L) were considered to be diabetic. Then they were randomly divided into 4 groups of 7 animals per group: i) diabetic control group (ZDF group; *n* = 7):received vehicle (saline) s.c. per day; ii) diabetic group treated with insulin (INS group; *n* = 7):administered long-lasting insulin (insulin glargine, Sanofi, France) s.c.per day; iii) diabetic group treated with DPP-4 inhibitor saxagliptin (SAXA group; *n* = 7): given saxagliptin (AstraZeneca, London, England) by using intragastric gavage at the dosage of 10 mg/kg/24 h; iv) diabetic group treated with *Glp1r* agonist liraglutide (LIRA group; *n* = 7): injected liraglutide (Novo Nordisk, Copenhagen, Denmark) subcutaneously at the dose of 200μg/kg/12 h. The dosages of the above two drugs are as described previously [9, 10]. Blood glucose, body weight and food intake were monitored simultaneously every week, meanwhile blood samples were collected from the tail veins and plasma glucose concentrations were estimated by a glucose meter (ACCU-CHEK Active, Roche Diagnostics, Basel, Switzerland). At the end of 20 weeks, the rats were anesthetized with 2.5% pentobarbital sodium and the bold samples were collected from left ventricle for the measurements of plasma glucose, total cholesterol (TC), triglycerides (TG), low-density lipoprotein cholesterol (LDL-c), high-density lipoprotein cholesterol (HDL-c), calcium, and phosphate, utilizing an automatic biochemical analyzer (Aeroset, American). Besides, glycated hemoglobin (HbA1c) levels were detected in serum sample using VARIANT TURBO instrumentation (Bio-Rad). Alkaline phosphatase, liver/bone/kidney (*Alpl*), osteocalcin (*Ocn*), procollagen1 N-terminal peptide (*P1NP*), C-terminal telopeptide of collagen type 1 (*CTX*) were determined by mouse enzyme-linked immunosorbent assay (ELISA) (Uscnlife, Wuhan EIAab Science Co, Ltd., Wuhan, China).

### Analysis of bone structure

Analysis of the trabecular bone architecture was carried out in a 2.5-mm-thick region, 1.2 mm distal to the growth plate of the knee joint. All specimens were analyzed at a 20 μm nominal resolution with a SHARP micro-Computed Tomography (micro-CT) scanner and the associated analysis software (ZKKS-MCT, SHARP, Japan). Images were reconstructed based on Feldkamp convolution back-projection algorithm and segmented into binary images (8-bit BMP images) using adaptive local thresholding. The same thresholding was applied to the images to separate the trabecular bones from the background, as described previously [11]. The parameters including volumetric bone mineral density (BMD), percent bone volume with respect to trabecular volume, trabecular thickness, trabecular separation, trabecular number, structure model index, and cortical thickness were measured.

### Oral glucose tolerance test (OGTT)

After a 12 h overnight fast and with free access to water provided, the rats in each group were given glucose at a concentration of 2 g/kg of body weight via intragastric gavage,without anesthesia. Blood samples which were collected from the tail vein were taken at 0 (prior to glucose concentration), 30, 60, 90 and 120 min. Blood glucose area under curve (AUC) is calculated from the area between 0 h and 2 h in the OGTT curve.

### Food consumption evaluation

Daily food intake is calculated by the difference between the amount of food provided and the amount of food left over after 24 hours, displayed in gram (g) and energy [12].

### Biomechanical testing

After slowly thawed, the left femur was placed in a material testing machine (InstronElectroPuls, E1000, USA) on two supports separated by a distance of 20 mm. At the same time, a three-point bending test was performed by applying a load at the midpoint of the right femoral diaphysis whose biomechanical quality was measured at a loading speed of 2 mm/min. When the central loading point was displaced, the load and the displacement were recorded until the specimen was broken. From the recorded resulting forces and displacements, structural properties included maximum load (ultimate strength), maximum displacement, stiffness (the linear part of the curve representing the slope of the elastic deformation), and energy absorption (area under the curve) were calculated based on the load-deformation curve.

### AGEs-BSA preparation

AGEs-bovine serum albumin (AGEs-BSA) was prepared according to previously reported [11]. AGEs-BSA was produced by incubation of100mg/ml BSA with100mM ribose in 150 mM phosphate buffered saline (PBS), under sterile conditions, pH 7.4 at 37 °C for 3 weeks. Unincorporated sugars were removed by PD-10 column chromatography and dialysis against phosphate-buffered saline. Control non-glycated BSA was incubated in the same conditions except for the absence of reducing sugars. The AGEs-BSA was passed through a Detoxi-Gel column to remove contaminated endotoxin. Endotoxin levels in the preparation were determined with the amebocyte lysate assay kit and were found to be below 0.25 EU/ml. The successfully prepared AGEs were assessed by their characteristic fluorescence-emission maximum at 420 nm upon excitation at 340 nm.

### Mature osteoblast derived from mouse long bone

Two one-month-old C57 mice were sacrificed. The bones were cut from isolated femurs and tibiae, digested with 0.8 ml collagenase and then got population for 4 times. Then it was inoculated into a culture medium, which was then incubated with α-MEM and 10%FBS medium at 37 °C in a humidified atmosphere of 5% CO_2_. AGEs, GLP-1 or exendin were added to the culture. Cultures were grown for 8 days with half medium changes twice daily.

### MTT assay

The cells were seeded in 96-well plates. After experimental treatments, following by 150 μl DMSO, colorimetric 3-(4, 5-dimethylthiazol-2-yl)-2, 5-dihenyltetrazolium bromide MTT solution was added to each well. Absorption was measured at 570 nm with a microplate reader. The cell injury was determined by the absorbance measured by MTT assay.

### Alkaline phosphatase

The cells were rinsed with PBS and then fixed in 10% formalin at room temperature for 15 min. After the fixed solution was removed, the cells were washed with D.I. water. Adding the 1-Step NBT/BCIP Solution (Thermo Scientific), the staining process lasted for up to 1 hour under monitoring. The staining solution was aspirated and the cell layer was washed with D.I. water three times followed by air dry.

### Immunofluorescent staining

After treatment, the cells were washed twice with PBS and fixed with 3.7% formalin solution for 10 min at room temperature. Then, the cells were permeabilized with 0.5% Triton X-100 in PBS for 10 min and blocked with 3%BSA in PBS for 1 h. After incubation with the specific primary antibody (1:200) overnight at 4 °C, slides were washed 2 times with PBS Tween-20 (PBST) for 10 min each. Secondary fluorescence antibodies (1:300) in PBST were incubated for 1 h at room temperature and nuclei were subsequently stained with DAPI. Utilizing the laser confocal microscope (FV10-ASW, Olympus, Tokyo, Japan) to captured the images.

### RT-PCR for detection of different markers

Total RNA was extracted from osteoblasts and right tibia after treatments, using TRIZOL.

reagent. Reverse transcription was conducted with 0.8 μg of total RNA using the PrimeScript one-step RT-PCR kit. Real-time PCR was carried out in a Real-Time PCR System (Stratagene/Agilent Technologies, Wilmington, DE, USA) using SYBR Premix Ex TaqII. The cycling conditions were as follows: 95 °C for 2 min and 40 cycles of 95 °C for 5 sec, 60 °C for 30 sec [11]. For each rat, the gene expression was normalized with that of the housekeeping gene *Gapdh* and expressed as 2^-ΔΔCt^. The following primers were used [13, 14]: *Gapdh* region sense: AGACAGCCGCATCTTCTTGT and region antisense: TGATGGCAACAATGTCCACT; *Osx* region sense: GGCTTTTCTGTGGCAAGAGGTT and region antisense: CGCTGATGTTTGCTCAAGTGGTC; *Alpl* region sense: CCAGAAAGACACGTTGACTGTGG, and region antisense: TCTTGTCCGTGTCGCTCACCAT; *Ocn* region sense: AGCTCAACCCCAATTGTGAC and region antisense: TCCTGGAGAGTAGCCAAAGC; *Opn* region sense: GCTAAGCCTCAGCATCCTTG and region antisense: AAGCAAACCACTGCCAGTCT; *Rage* region sense: ACAGAAACCGGTGATGAAGG, and region antisense: ATTCAGCTCTGCACGTTCCCT.

### Western blot analysis for detection of different markers

After one night of starvation in serum-free medium, the osteoblasts were stimulated for 3 days under the above culture conditions. Proteins were then extracted from the osteoblasts using RIPA lysis buffer. The primary antibodies were rabbit anti-*Glp1r* IgG (1:500), rabbit anti-*Rage* IgG (1:500), mouse anti-*Gapdh* IgG (1:1000), rabbit anti-*Gapdh* IgG (1:1000), rabbit anti-*Cyclophilin B* IgG (1:1000), and the secondary antibody was goat HRP-conjugated IgG (1:1000) and rabbit HRP-conjugated IgG (1:1000). The blots were cut prior to hybridisation with antibodies during blotting, and the ECL chemiluminescence detection system was used to determine the bands.

### Determination of ROS generation

The flow cytometry and the probe DCFH-DA were used to detect the intracellular ROS production. Osteoblasts were grown in 10 cm plates and simultaneously subjected to various culture conditions as described above for 2 hours. Afterwards, control medium containing with 10 μM DCFH-DA replaced the medium, which then was incubated in the dark for 30 minutes. Intracellular ROS generation was observed under a fluorescent microscope (Olympus, Tokyo, Japan). The flow cytometer was used to determine the DCF fluorescence, and the data were normalized to the control values.

### Statistical analysis

Data were expressed as mean ± SD and analyzed using SPSS for Windows version 15.0 (SPSS Inc., Chicago, IL, USA). One-way ANOVA followed by the Newman-Keuls test or Dunnett’s T3 (equal variances not assumed) was performed for multiple comparisons. Differences at *P* < 0.05 were considered to be significant.

## Results

### Metabolic parameters in diabetic rats

**Fig. 1:**
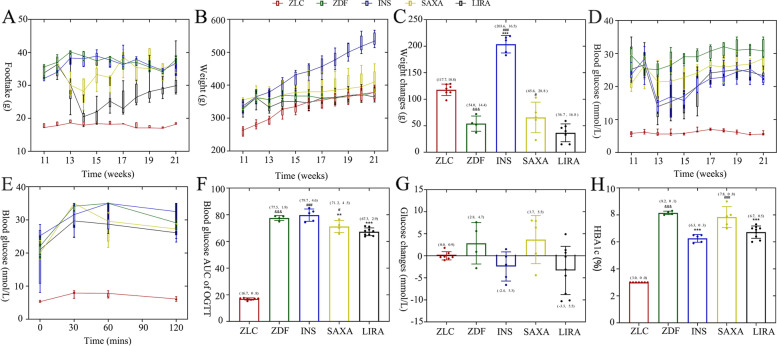
Metabolic parameters in ZDF rats. **A** Foodtake throughout the study period; **B** Body weight throughout the study period; **C** Body weight changes (at Week 21 from Week 11); **D** Blood glucose throughout the study period; **E** Blood glucose profiles during the OGTT at the end of 9 weeks treatment; **F** AUC of the blood glucose profiles during the OGTT at the end of 9 weeks treatment; **G** Blood glucose changes (at Week 21 from Week 11); **H** HbA1c at the end of 9 weeks treatment. 11-week-old ZDF rats were randomly assigned into four subgroups: treated with vehicle (ZDF); treated with insulin (INS); treated with saxagliptin (SAXA); treated with liraglutide (LIRA). OGTT, oral glucose tolerance test; HbA1c, glycated hemoglobin; AUC, area under curve. The Newman-Keuls Multiple Comprison Test was used, and (mean, SD) was displayed above the bar. ^&^*P* < 0.05, ^&&^*P* < 0.01, ^&&&^*P* < 0.001 vs. ZLC group; *P < 0.05, ** *P* < 0.01, *** *P* < 0.001 vs. ZDF group; ^#^*P* < 0.05, ^##^ *P* < 0.01, ^###^ *P* < 0.001 vs. LIRA group

The food intake of ZDF control rats was increasing every week, while a decreasing trend of weekly food intake was observed in the ZDF rats treated with liraglutide (Fig. 1A). The body weights of ZDF rats were heavier compared to lean rats at all the time points (*P* < 0.001) (Fig. 1B). Insulin significantly increased the weight of ZDF rats. It is noticeable that the weight of both untreated ZDF rats and ZDF rats treated with liraglutide decreased to the same level as the lean group (Fig. 1B). However, the weight did not decrease in the insulin and saxagliptin treated rats (Fig. 1C).

Furthermore, ZDF rats exhibited an elevated blood glucose compared to lean controls (Fig. 1D). After the treatment of insulin, saxagliptin or liraglutide, the glucose AUC of.

OGTT demonstrated impaired glucose tolerance in the ZDF, INS group, SAXA group and LIRA group. (Fig. 1E, F) Insulin and liraglutide decreased fasting blood glucose (FBG) (Fig. 1E) and HbA1c level at the end of the 20th week (*P* < 0.001) (Fig. 1H).

### Liraglutide ameliorated bone remodeling parameters of ZDF rats

**Fig. 2:**
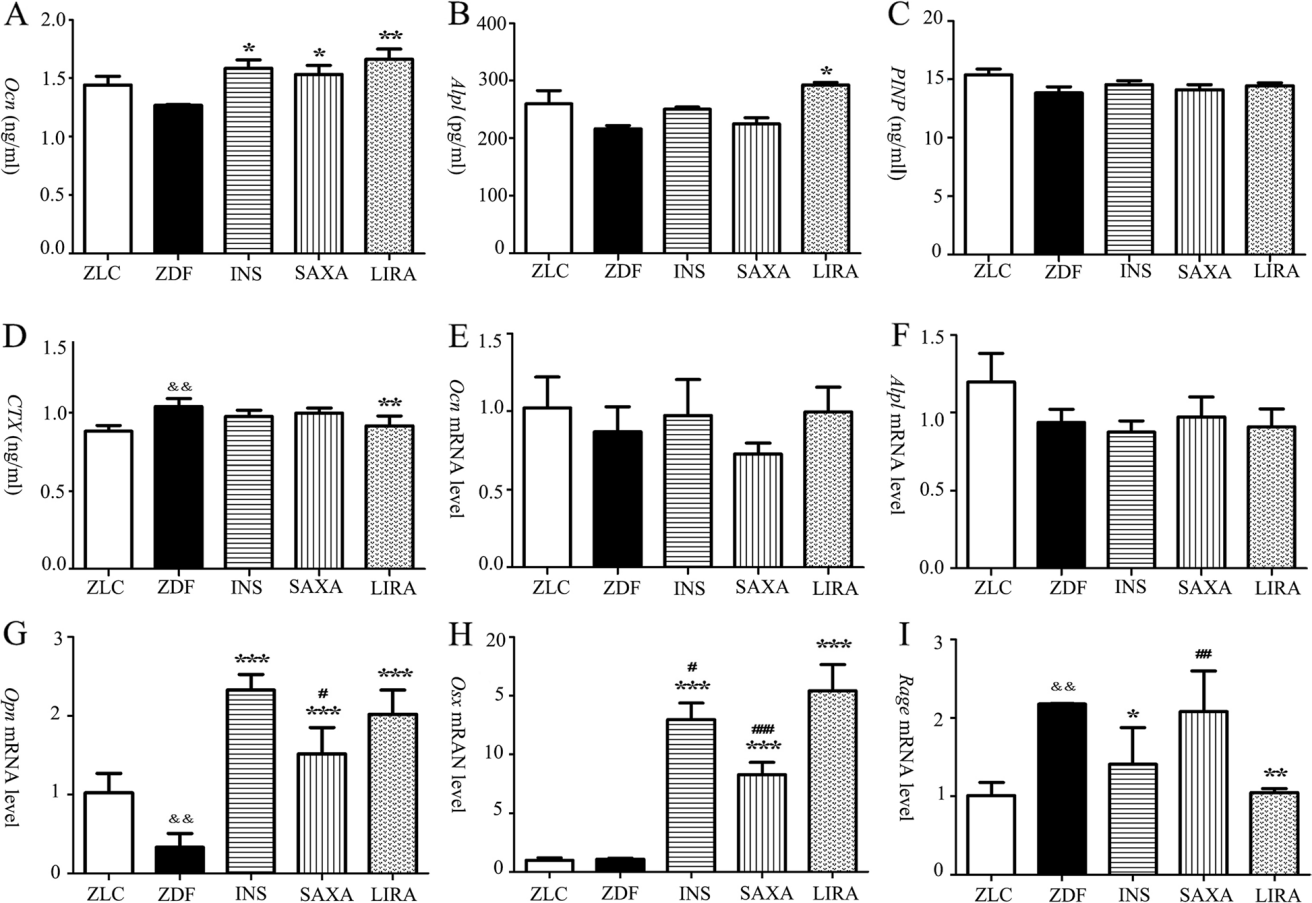
Effects of 9 weeks treatment on serum and bone remodeling parameters. **A-D** Serum bone remodeling parameters; **E-H** mRNA levels of bone remodeling parameters in femur; **(I)** mRNA levels of *Rage* in femur. *Ocn*, osteocalcin; *Alpl*, bone alkaline phosphatase; *P1NP*, procollagen type 1 N-terminal peptide; *CTX*, C-terminal telopeptide of collagen type 1; *Opn*, osteopontin; *Osx*, Osterix; *Rage*, receptor for AGEs. Values are means ± SD (*n* = 7). The Newman-Keuls Multiple Comprison Test was used. ^&^*P* < 0.05, ^&&^*P* < 0.01, ^&&&^*P* < 0.001 vs. ZLC group; *P < 0.05, ** *P* < 0.01, *** *P* < 0.001 vs. ZDF group; ^#^*P* < 0.05, ^##^ *P* < 0.01, ^###^ *P* < 0.001 vs. LIRA group

Bone remodeling parameters were listed in Fig. 2**A-I**. The serum concentration of *Ocn* was significantly increased after treatment of insulin, saxagliptin and liraglutide (Fig. 2A). Liraglutide increased the serum activity of *Alpl* in ZDF rats. However, similar changes could not be observed in saxagliptin group (Fig. 2B). No statistically significant change in serum *P1NP* level was noticed (Fig. 2C). *CTX* was decreased significantly after treatment with liraglutide (Fig. 2D).

Besides, biochemical markers in femur were also detected (Fig. 2E-H). The treatment of insulin, saxagliptin, and liraglutide could reverse the decreased expression of *Opn*. Significant increases in *Osx* levels were observed in ZDF rats after in three groups.

### Liraglutide affected the gene expressions of ***rage***

The expression of *Rage* in transcriptional level was detected after insulin, saxagliptin or liraglutide for 9 weeks (Fig. 2I). The expression of *Rage* mRNA in ZDF group significantly increased compared to the lean group (*P* < 0.05). Liraglutide and insulin reduced the expression of *Rage* to a level comparable to lean controls.

### Liraglutide improved bone trabecular microarchitecture of ZDF rats

**Fig. 3:**
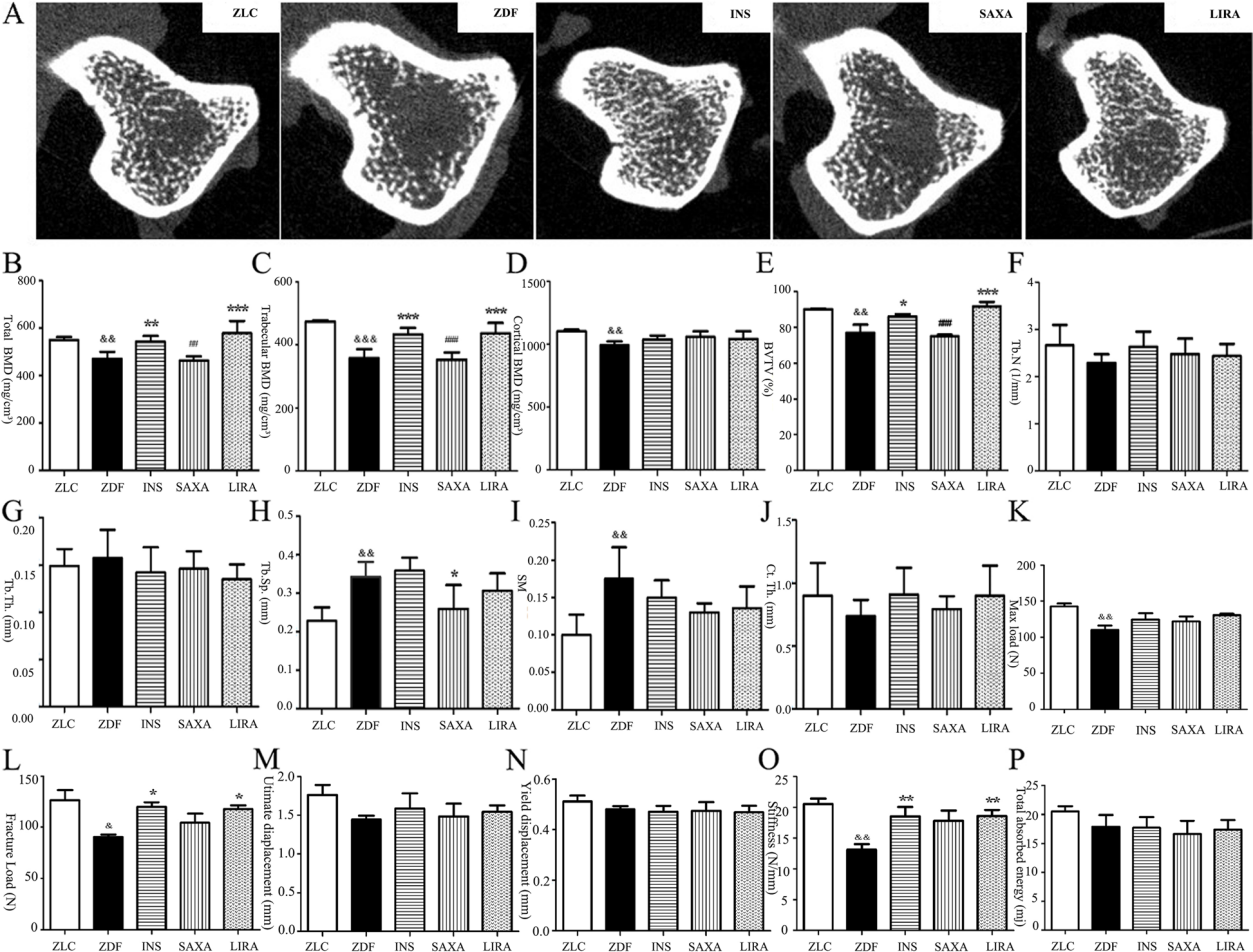
Bone effect of ZDF rats after treatment of Liraglutide. **A-I** The distal femur of structural bone parameters analyzed by micro-CT; **B** Total bone mineral density (BMD); **C** trabecular BMD; **D** cortical BMD; **E** Bone volume per total volume (BV/TV); **F** Trabecular number (Tb.N); **G** Trabecular thickness (Tb.Th.); **H** Trabecular spacing (Tp.Sp.); **I** Structure model index; **J** Cortical thickness (Ct.Th.); **K-P** Femoral biomechanical structural properties in ZDF rats via three-point bending test, including max load, fracture load, ultimate displacement, yield displacement, stiffness, and total absorbed energy. Values are means ± SD (*n* = 7); The Newman-Keuls Multiple Comprison Test was used. ^&^*P* < 0.05, ^&&^*P* < 0.01, ^&&&^*P* < 0.001 vs. ZLC group; *P < 0.05, ** *P* < 0.01, *** *P* < 0.001 vs. ZDF group; ^#^*P* < 0.05, ^##^ *P* < 0.01, ^###^ *P* < 0.001 vs. LIRA group

As displayed by the micro-CT scans, ZDF rats exhibited fewer, thinner and more broken trabecular bones, as well as lower BMD in femur compared with the rats in the normal control group (Fig. 3A-J). The trabecular architecture was remarkably improved in the liraglutide- and insulin-treated ZDF rats compared with the control ZDF rats.

### Liraglutide increased the bone strength of ZDF rats

Three-point bending assessment of cortical bone revealed that ZDF rats presented with the lower mechanical responses, including max load, fracture load and stiffness (Fig. 3K-P). Liraglutide treatment resulted in a significant increase in both fracture load (*P* < 0.05, + 70.5%) and stiffness (*P* < 0.01, + 38.3%). However, no significant difference was observed in max load, ultimate displacement, yield displacement and total absorbed energy between the liraglutide group and ZDF group.

### GLP-1 attenuated AGEs-mediated damage in the proliferation of osteoblasts

**Fig. 4:**
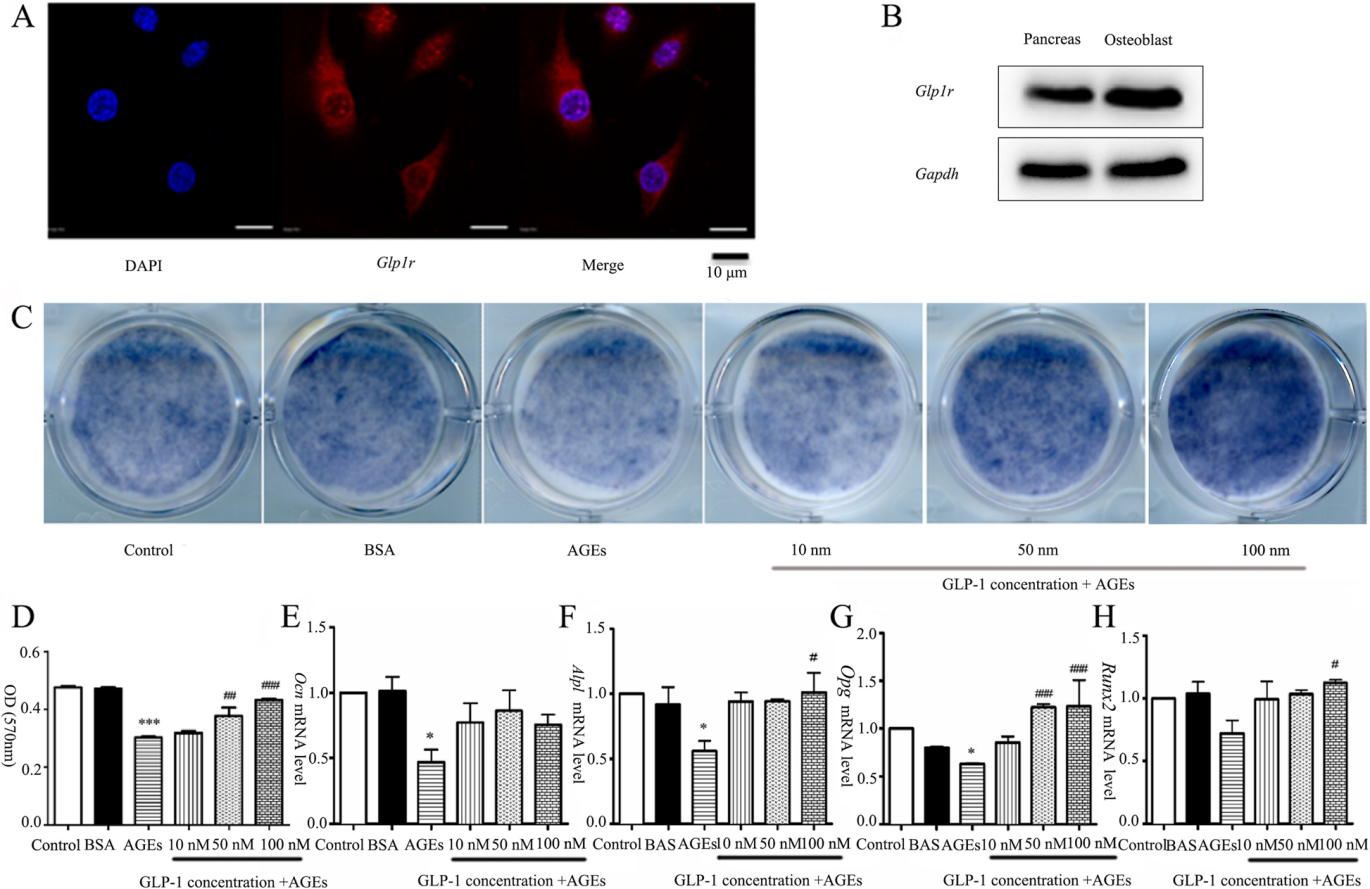
Effect of GLP-1 on the AGEs-mediated damage in proliferation and differentiation of osteoblasts. **A, B** *Glp1r* protein was detected by immunofluorescence and western blot; **C** *Alpl* staining, which indicates the early stage of osteoblastogenesis was performed at the indicated time points; **D** Cell proliferation was measured by the absorbance at 570 mm with a MTT assay; **E-H.** The mRNA expression of osteogenic differentiation markers (*Ocn*, *Alpl*, *Opg*, *Runx2*) of osteoblasts assessed by real-time PCR. *Ocn*, osteocalcin; *Alpl*, Alkaline phosphatase, liver/bone/kidney; *Opg*, osteoprotegerin; *Runx2*, Runt-related transcription factor 2. Values are means ± SD from three independent experiments. The Newman-Keuls Multiple Comprison Test was used. *P < 0.05, **P < 0.01, ***P < 0.001 vs. control group; ^#^*P* < 0.05, ^##^*P* < 0.01, ^###^*P* < 0.001 vs. AGEs group

*Glp1r* protein was presented in the long bone-derived osteoblasts detecting by both immunocytochemistry (Fig. 4A) and western blot (Fig. 4B). The osteoblasts were induced by control medium, BSA(200 μg/mL) and AGEs(200 μg/mL) in the absence or presence of the indicated concentration of GLP-1, which was renewed once every day. Osteogenic proliferation was evaluated by MTT assay (Fig. 4D). The OD value (0.30 ± 0.01) in AGEs group was significantly lower than the control group (0.48 ± 0.01) (*P* < 0.001). After treatments with 50 and 100 nM GLP-1, the OD value significantly increased than that in AGEs group. These results indicated that GLP-1 not only reversed the osteogenesis inhibitory effect of AGEs, but also ameliorated osteoblasts proliferation in a dose-dependent manner.

### GLP-1 attenuated AGEs-mediated damage in the differentiation of osteoblasts

There was an obvious increase in *Alpl* staining after GLP-1 was added to mice osteoblasts that undergoing induced osteoblastogenic differentiation for 7 days (Fig. 4C). As shown in Fig. 4E-H, the transcriptional levels of *Ocn*, Alp, and *Opg* were all decreased after exposure to AGEs. Pre-treatment of osteoblasts with 100 nM GLP-1 led to the increased mRNA expressions of *Ocn*, *Alpl*, *Opg*, and *Runx2* compared with the AGEs group.

### GLP-1 attenuated AGEs-mediated oxidative stress in osteoblasts partly through the RAGE pathway

**Fig. 5:**
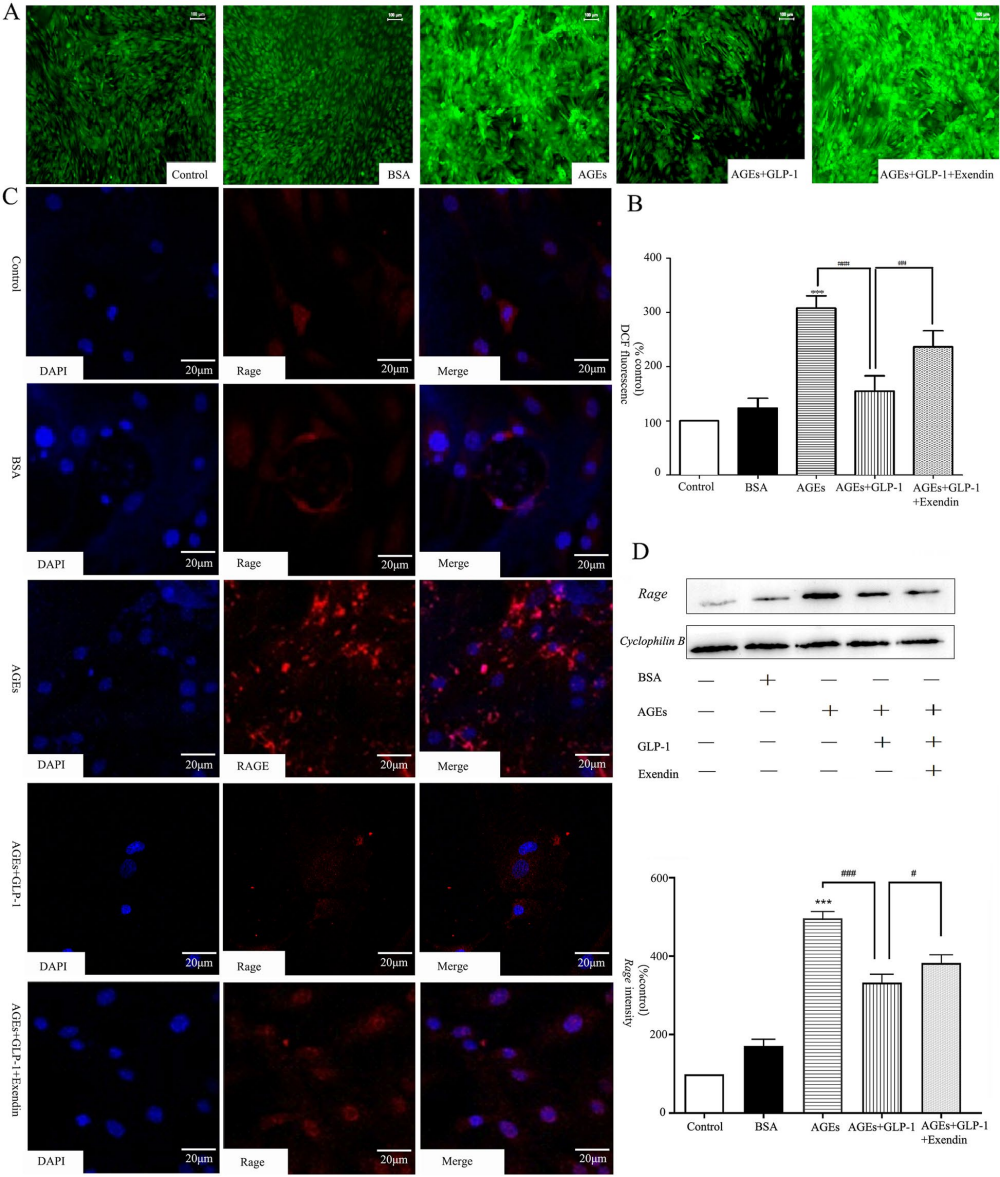
Effect of GLP-1 on the AGEs-mediated ROS and *Rage* expression. **A, B** Intracellular ROS generation was measured with the probe DCFH-DA, and visualized using a fluorescent microscope; **C** Immunofluorescence was used to detect *Rage* antibody (red fluorescence) coupled to secondary antibodies. Nuclei were stained with DAPI (blue fluorescence); **D** *Rage* Protein and mRNA relative levels in osteoblasts were detected by western blot analysis and PCR. Values are means ± SD from three independent experiments. The Newman-Keuls Multiple Comprison Test was used. *P < 0.05, **P < 0.01, ***P < 0.001 vs. control group; ^#^*P* < 0.05, ^##^*P* < 0.01, ^###^*P* < 0.001 vs. AGEs group

Oxidative stress was examined by fluorescence microscopy. DCF fluorescence of osteoblasts exposed to AGEs was strikingly increased compared with the control group (*P* < 0.001) (Fig. 5A, B). DCF fluorescence of the AGEs+GLP-1 group was significantly lower than that of the AGEs group(*P* < 0.01). After adding Exendin (*Glp1r* blockers), DCF fluorescence was recovered and significantly higher than that of the AGEs+GLP-1 group (*P* < 0.01).

After treatment with AGEs for 96 h, the fluorescence intensity of *Rage* was strengthened and significantly higher than the BSA group. After GLP-1 treatment, the fluorescence intensity of *Rage* was obviously decreased, and this could be reversed by the addition of Exendin (Fig. 5C). Consistently, both the *Rage* mRNA and proteins in osteoblasts were significantly higher in the AGEs group than those in the control group. GLP-1 significantly down-regulated the *Rage* expression. Exendin, again, could reverse the effects of GLP-1 on the expression of *Rage* in both transcriptional and translational levels (Fig. 5D).

## Discussion

GLP-1 plays a vital role in bone turnover [15]. In an animal experiment, GLP-1 or exendin-4 was used to treat alone to reversed the decrease of bone in femurs and vertebrae in hyperlipidemia and hypercaloric Wistar rats [16]. However, in a clinical trial, T2DM patients treated with metformin were concurrently received either exenatide or insulin glargine, only the exenatide group lost body weight significantly and had maintained bone mineral density levels after 44 weeks of treatment [17], which is due to weight loss [18], and insulin administration may contribute to reduction in bone resorption [19]. These results suggest that exenatide may promote BMD as well as weight reduction compared with insulin. Otherwise, in an open-label, randomized, controlled trial, comparing the effect of 37 perimenopausal non-diabetic women treated with 1.2 mg liraglutide once daily with no treatment, the systemic and peripheral bone mineral content (BMC) in the treated group was significantly higher than the untreated group. Liraglutide increased the bone formation marker (*P1NP*) without concomitant increase in bone resorption markers (*CTX*) [3]. Nevertheless, the mechanism of GLP-1 on bone turnover in type 2 diabetes remains unknown. In our study GLP-1 was demonstrated to attenuate the AGEs-mediated damage to osteoblast proliferation and differentiation through the RAGE pathway in vitro.

Studies illustrated that *Glp1r* was expressed in mature cells, such as MC3T3-E1 cells [20], osteocyte-like MLO-Y4 cells [21]. We focus on mature osteoblasts derived from mouse long bone, which represent bioactivity of osteoblast in vivo better. We proved that *Glp1r* existed in the mature murine osteoblasts by immunocytochemistry and western blot. GLP-1 and its receptor agonist can regulate bone metabolism directly because of the expression of *Glp1r* in osteoblasts.

Liraglutide exerted anabolic effects on the skeleton in non-obese spontaneous diabetic rats [22]. However, there are few pieces of evidence to evaluate the effects of GLP-1 in obese diabetic models. As a common animal model of type 2 diabetes, ZDF rats exhibit the same insulin resistance and reduced insulin production as human patients [23]. Previous studies have detected lower bone formation markers and higher bone resorption markers in ZDF rats [24], which were consistent with our results. After treatment of liraglutide, significant attenuation of trabecular bone loss and marked improvement of the trabecular bone structure were observed, which can be attributed to the conservation of trabecular number and BMD. Furthermore, the ultimate load to fracture increased significantly in the femora of liraglutide-treated compared to the untreated diabetic animals, indicating an increase in bone density and strength. Generally, diabetic subjects who gained weight had slightly higher bone mass than non-diabetic subjects during the initial weight gain phase of diabetes, however, in the present study, as displayed by the micro-CT scans, ZDF rats exhibited fewer, thinner and more broken trabecular bones, as well as lower BMD in femur compared with the rats in the normal control group. This is due to patients developed severe osteoporosis and bone quality decreased significantly in the late. Therefore, in this study, bone quality was determined after the rats were sacrificed at the 21st week, when the progress was already in the stage of osteoporosis.

Saxagliptin is a highly effective inhibitor of dipeptidyl peptidase 4. Through selective inhibition of dipeptidyl peptidase 4, endogenous GLP-1 is increased and the level of glucose-dependent insulin-releasing polypeptides is upregulated to regulate blood glucose [25]. Previous studies have shown that saxagliptin can improve bone metabolism in patients with T2DM, promote bone formation and improve various serum bone metabolism indicators [4, 26]. It is strange that the effect of saxagliptin treatment on bone remodeling, trabecular structure and strength was significantly lower than that of GLP-1 agonist, which is due to the complexity of DPP4 inhibitors, which can increase the concentration of GLP-1 to some extent, but affect the secretion of GLP-1 and *Glp1r* activity [27, 28].

Biochemical markers were detected to explore the underlying mechanisms of GLP-1’s anti-osteoporotic effect in vivo. *Alpl*, type 1 collagen (*COL 1*) and *Ocn* are common markers of bone formation [29]. As shown in the previous study, all of the above markers are up-regulated. In old rats with OVX-induced osteoporosis, the levels of *Alpl*, *COL 1* and *Ocn* mRNA were increased after 16 weeks of exendin-4 administration [30]. The same result was found in the femurs of GK rats treated with liraglutide [22]. At the same time, in femurs of liraglutide-treated GK rats, mRNA expression of *Runx2*, a key transcriptional factor that can stimulate the expression of other osteoblast-specific genes during the early stage of osteogenesis [31], was increased along with mRNA for other bone formation markers, such as *Alpl*, *COLL*, *Ocn*, and *Opg*, compared with vehicle-treated GK rats [15]. In the present study, we demonstrated that liraglutide could significantly increase serum *Alpl* and *Ocn*, along with mRNA expressions of *Opn* and *Osx* in femurs, which indicated that GLP-1 may effect on the various stages of bone formation. In addition, in a recent study, *CTX* decreased during 24 weeks of treatment with the GLP-1 receptor agonist liraglutide [32]. Also, we found that liraglutide decreased *CTX* (a bone resorption marker), suggesting that GLP-1 may regulate bone resorption. Although these findings did not indicate whether the effect is progressive or parallel, it has been shown that GLP-1 does have a protective effect on osteoblasts.

The formation and accumulation of AGEs is considered to be one of the main causes of osteoblast activity inhibition and bone quality damage caused by diabetes. AGEs are formed by the Maillard process which is a non-enzymatic reaction among ketones or aldehydes, amino proteins groups, lipids and nucleic acids which contributes to the aging of macromolecules [33–35]. It has been demonstrated that the accelerated accumulation of AGEs was associated with the decline of BMD in the bone collagen of streptozotocin-induced diabetic rats [36]. AGEs are formed on type 1 collagen, which a major matrix protein of bone, also play a role in the decrease BMD in patients with diabetes [37]. Indeed, when rat osteoblastic cells were cultured on AGEs-modified type 1 collagen, *Alpl* activity and osteocalcin secretion were decreased and nodule formation was dramatically inhibited [36].

To further explore the mechanisms underlying the GLP-1-mediated bone formation, we also observed the effect of GLP-1 on AGEs-mediated osteoblast proliferation and differentiation. We discovered that the transcriptional expression level of *Ocn*, *Alpl*, *Opg*, and *Runx2* were all increased compared with AGEs in GLP-1-treated osteoblasts. These results demonstrated that GLP-1 ameliorated AGEs-mediated osteoblasts proliferation damage in a dose-dependent manner. Moreover, there was an obvious increase in *Alpl* staining when GLP-1 was added to mouse osteoblasts undergoing induced osteogenic differentiation. These data indicate that GLP-1 inhibits AGEs-induced damage in osteogenic proliferation and differentiation.

There is more and more evidence showing AGEs and their receptor (*Rage*) system induce oxidative stress, which in turn evokes inflammatory responses in vascular wall cells, osteoblasts and osteoclasts, thereby being involved in vascular calcification and osteoporosis in diabetes [38]. In *Rage* knockout mice, the number of osteoclasts and bone resorption decreased, bone mass and biomechanical strength were improved. In vitro-differentiated *Rage*-deficient osteoclasts exhibited that destroyed actin ring and sealing zone structures, damaged maturation, and decreased bone resorptive activity [39]. These observations suggest that *Rage* is involved in the recombination, adhesion and activation of osteoclast actin, which contributes to the reduction of bone mass in diabetes.

AGEs-RAGE interaction induces the generation of ROS through NADPH oxidase, which leads to osteoblast apoptosis [40, 41] and inhibits osteoblast proliferation and differentiation [5]. AGEs-mediated ROS and *Rage* expression were reduced after treatment with liraglutide for 9 weeks in the present study. In other terms, GLP-1 ameliorated osteoblasts ROS and it perhaps played a vital role in regulating the inherent antioxidant repair system, thus indirectly reducing intracellular ROS and preventing the accumulation of cellular damage. In the femur of the ZDF rats and their osteoblasts, we found that the expression of AGEs-modified albumin was up-regulated, resultantly inducing the differentiation of osteoblastic, while GLP-1 could reverse that, which suggested that GLP-1 may attenuate AGEs-mediated ROS damages in diabetes-associated osteoporosis through the RAGE pathway.

AGEs-mediated ROS damage may be affected by the ligation between Mammalian diaphanous 1 (DIAPH1, also known as mDia1) and *Rage*. As one of the most-characterized members of diaphanous-related formins family, mDia1 is a potent actin and microtubule polymerization factor, regulating many key cellular functions, such as cell adhesion, movement, cytokinesis, morphogenesis, cell polarity formation, and serum response factor activation [42–44]. Recent studies have identified that the formin homology (FH1) domain of mDia1 as a binding partner that interacts directly with the short cytoplasmic tail of *Rage* (C-terminal *Rage*, *ctRage*) [45]. Additionally, Nox4 functioned as the major catalytic component of endothelial NADPH oxidase and the main source of ROS production [46], which plays an important role in cell damage caused by oxidative stress. Using the Nox4 siRNA down-regulate the expression of Nox4 protein can effectively reduce the activity of NADPH oxidase, thus reducing the generation of ROS during oxidative stress [47, 48]. The expression of Nox4 increases during the differentiation and maturation of osteoblasts, and the formation of *Ocn* increases after Nox4 is activated, which can induce osteoporosis. It has been found that Nox4−/− mice have increased BMD, decreased the number of osteoblasts, and decreased the level of *Ocn* markers [49]. Meanwhile, it has been shown that mDia1 plays a key role in transducing the signal of AGEs upon ligation of *Rage* to activate Nox4 by recruiting Nox4 to the cell membrane. Therefore, the Nox4-derived ROS elevation triggers subsequent signaling pathways, and eventually results in endothelial hyperpermeability [50]. Therefore, we will further explore the mechanism of Nox4-derived ROS on bone injury in the future research.

## Conclusions

In conclusion, no previous report has compared the skeletal effects of insulin, liraglutide, and saxagliptin. This study prompted the indirect protective effects of *Glp1r* agonists on obese type 2 diabetic animals by promoting osteoblastogenesis and suppressing bone resorption. The mechanism of these effects may be partly mediated by AGEs-RAGE-ROS pathway via the interaction with *Glp1r*. This may provide benefits to therapy with liraglutide on diabetic osteoporosis as it could work as an agent against the AGEs–RAGE axis and may play a protective role against osteoporosis in diabetes. Thus, more large-scale human prospective studies are needed to evaluate the efficacy of *Glp1r* agonist or DPP-4 inhibitor in treating diabetic-associated osteoporosis.

## Supplementary Information

**Additional file 1: Supplementary Figure 1.** The original picture of Figure 3B (*Glp1r* protein was detected by western blot). **Supplementary Figure 2.** The original picture of Figure 3B (*Gapdh* protein was detected by western blot). **Supplementary Figure 3.** The original picture of Figure 4D (*Rage* Protein in osteoblasts were detected by western blot analysis). **Supplementary Figure 4.** The original picture of Figure 4D (*Cyclophilin B* Protein in osteoblasts were detected by western blot analysis). **Supplementary Figure 5.** The additionally original picture of Figure 4D (*Rage* Protein in osteoblasts were detected by western blot analysis). **Supplementary Figure 6.** The additionally original picture of Figure 4D (*Cyclophilin B* Protein in osteoblasts were detected by western blot analysis). **Supplementary Figure 7.** The protein marker (ThermoFisher, 26634).

## Data Availability

The datasets used and/or analysed during the current study are available from the corresponding author on reasonable request.

## Abbreviations

AGEs: Advanced glycation end products
*Alpl*: Alkaline phosphatase, liver/bone/kidney
AUC: Area under curve
BMD: Bone mineral density
BSA: Albumin from bovine serum
BV/TV: Bone volume per total volume
*COL 1*: Type 1 collagen
*COLL*: Collagen 1
Ct.Th.: Cortical thickness
*CTX*: C-terminal telopeptide of collagen type 1
CT: Computed Tomography
DAPI: 4′,6-diamidino-2-phenylindole
DCFH-DA: 2′,7′-dichlorodihydrofluorescein diacetate
DMEM: Dulbecco’s modified Eagle’s medium
DMSO: Dimethyl sulfoxide
DPP-4: Dipeptidyl peptidase-4
ELISA: Enzyme linked immunosorbent assay
FBG: Fasting blood glucose
FBS: Fetal bovine serum
GLP-1: Glucagon-like peptide-1
*Glp1r*: GLP-1 receptor
GLP1RA: *Glp1r* agonist
*Rage*/RAGE: Receptor for AGEs
IF: Immunofluorescence
HbA1c: Glycated hemoglobin
HDL-c: High-density lipoprotein cholesterol
HRP: horse radish peroxidase
LDL-c: Low-density lipoprotein cholesterol
MMT: 3-(4,5-dimethylthiazozyl)-2,5-diphenyl tetrazolium bromide
*Ocn*: Osteocalcin
*Opg*: Osteoprotegerin
*Opn*: Osteopontin
*Osx*: Osterix
OGTT: Oral Glucose Tolerance Test
*P1NP*: Procollagen1 N-terminal peptide
PBS: Phosphate buffered saline
PBST: PBS Tween-20
ROS: Reactive oxygen species
RT-PCR: Real Time Polymerase Chain Reaction
*Runx2*: RUNX Family Transcription Factor 2
SD: Standard Deviation
Tb.N: Trabecular number
Tb.Th.: Trabecular thickness
Tp.Sp.: Trabecular spacing
TC: Total cholesterol
TG: Triglycerides
WB: Western Blot
ZDF: Zucker Diabetic Fatty
ZLC: Zucker Lean Control
