# Supplementary material for: Glucagon-like peptide-1 attenuates diabetes-associated osteoporosis in ZDF rat, possibly through the RAGE pathway
Source: BMC Musculoskelet Disord. 2022 May 17;23:465. doi: 10.1186/s12891-022-05396-5 (PMC9112483; doi:10.1186/s12891-022-05396-5)
Supplement: Supplementary file 1 — Additional file 1: Supplementary Figure 1. The original picture of Figure 3B (Glp1r protein was detected by western blot). Supplementary Figure 2. The original picture of Figure 3B (Gapdh protein was detected by western blot). Supplementary Figure 3. The original picture of Figure 4D (Rage Protein in osteoblasts were detected by western blot analysis). Supplementary Figure 4. The original picture of Figure 4D (Cyclophilin B Protein in osteoblasts were detected by western blot analysis). Supplementary Figure 5. The additionally original picture of Figure 4D (Rage Protein in osteoblasts were detected by western blot analysis). Supplementary Figure 6. The additionally original picture of Figure 4D (Cyclophilin B Protein in osteoblasts were detected by western blot analysis). Supplementary Figure 7. The protein marker (ThermoFisher, 26634). [file 12891_2022_5396_MOESM1_ESM.zip › Supplementary Material 20220330.docx]

Supplementary Material


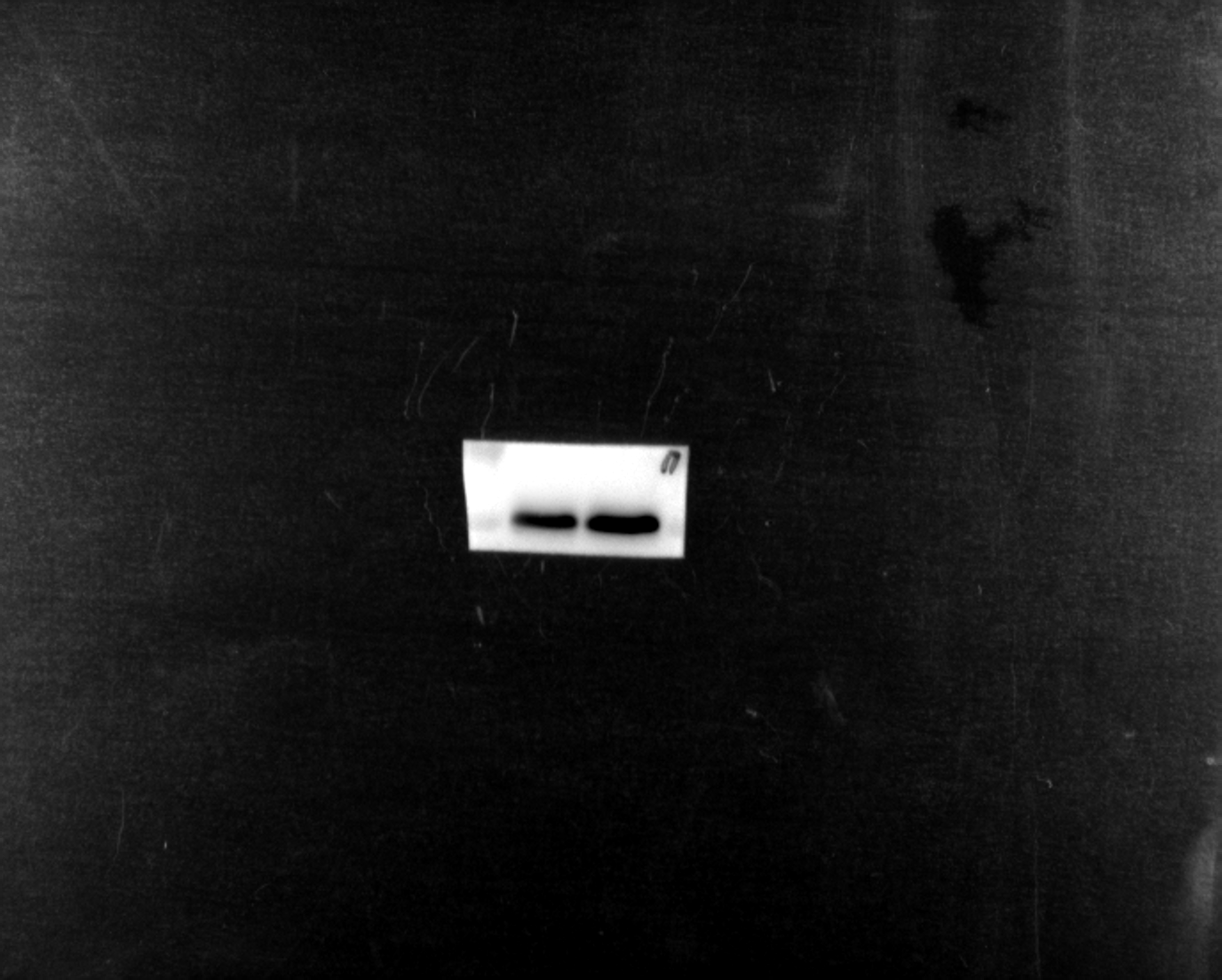


**53kDa**

**50kDa**

**70kDa**

**Supplementary Figure 1.** The original picture of **Figure 3B** (*Glp1r* protein was detected by western blot).

**36kDa**

**40kDa**

**35kDa**


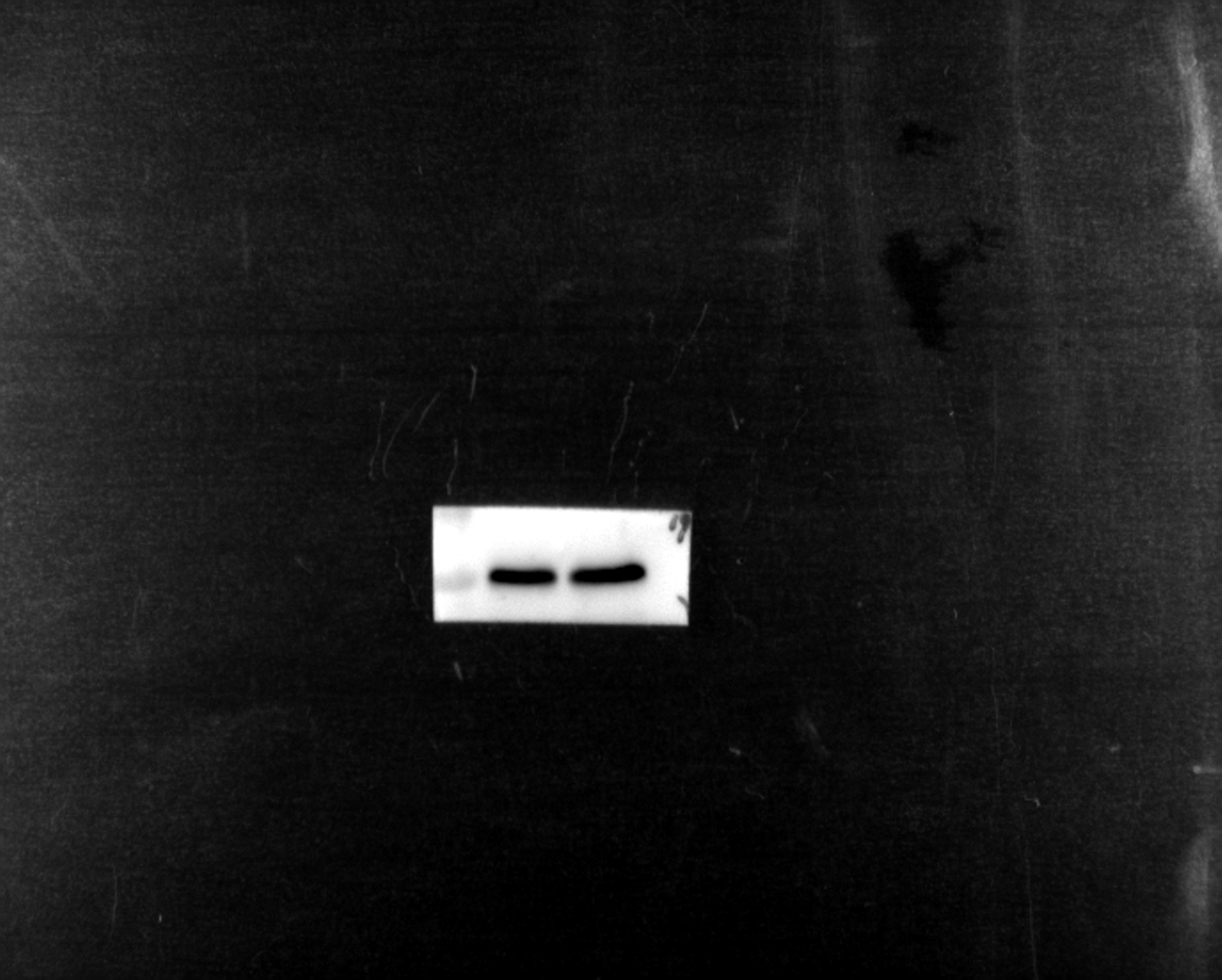


**Supplementary Figure 2.** The original picture of **Figure 3B** (*Gapdh* protein was detected by western blot).


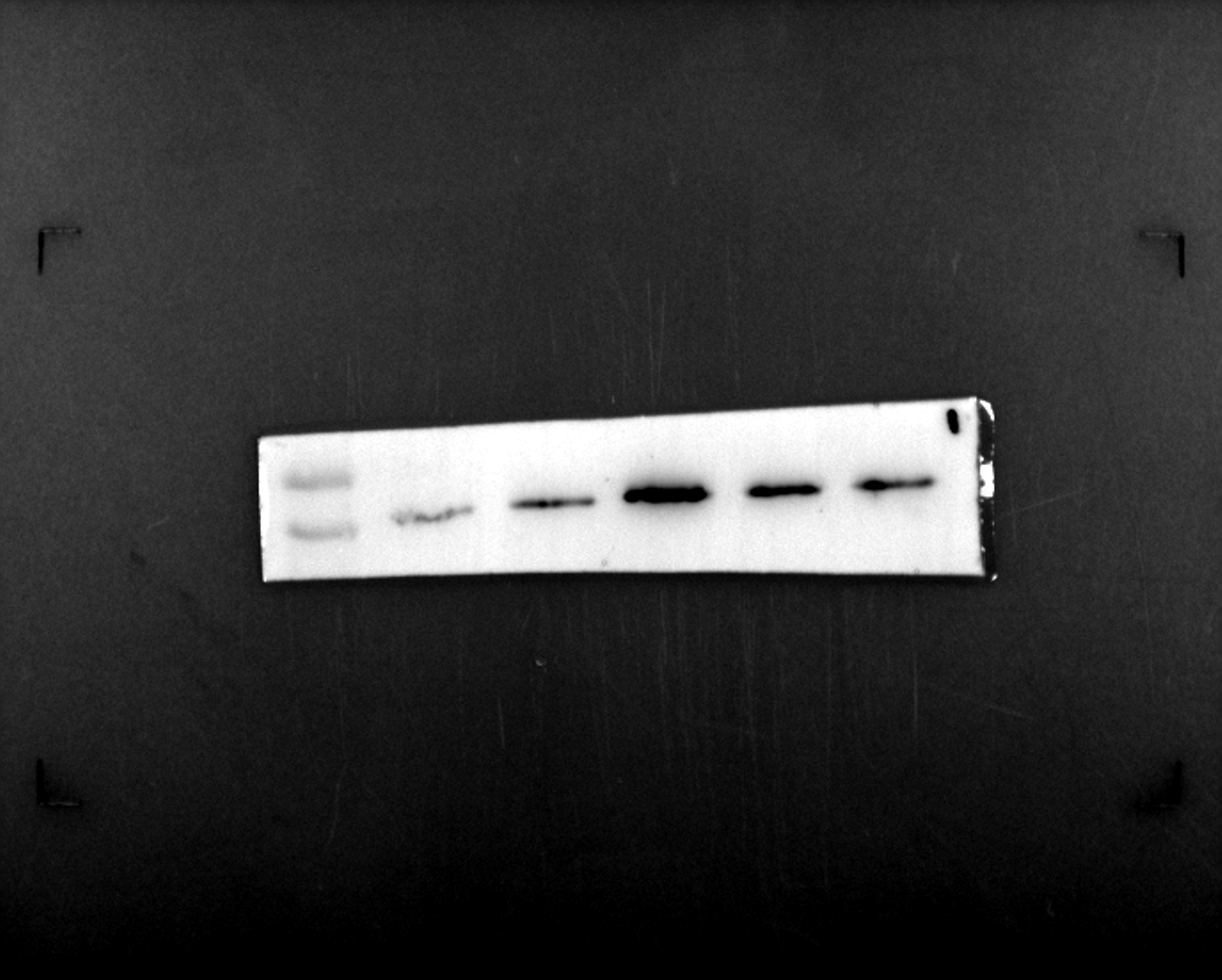


**40kDa**

**50kDa**

**43kDa**

**Supplementary Figure 3.** The original picture of **Figure 4D** (*Rage* Protein in osteoblasts were detected by western blot analysis).


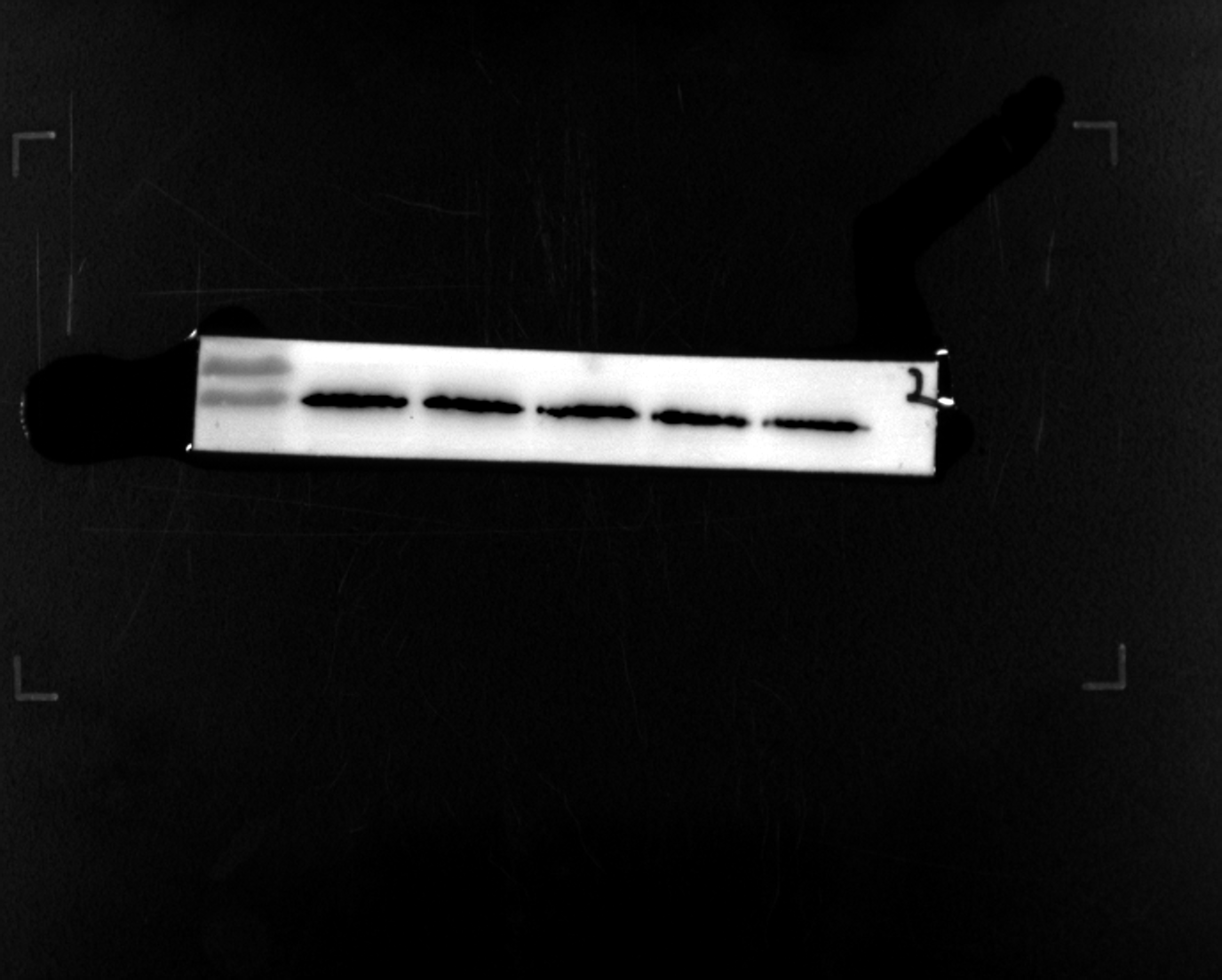


**25kDa**

**35kDa**

**23kDa**

**Supplementary Figure 4.** The original picture of **Figure 4D** (*Cyclophilin B* Protein in osteoblasts were detected by western blot analysis).


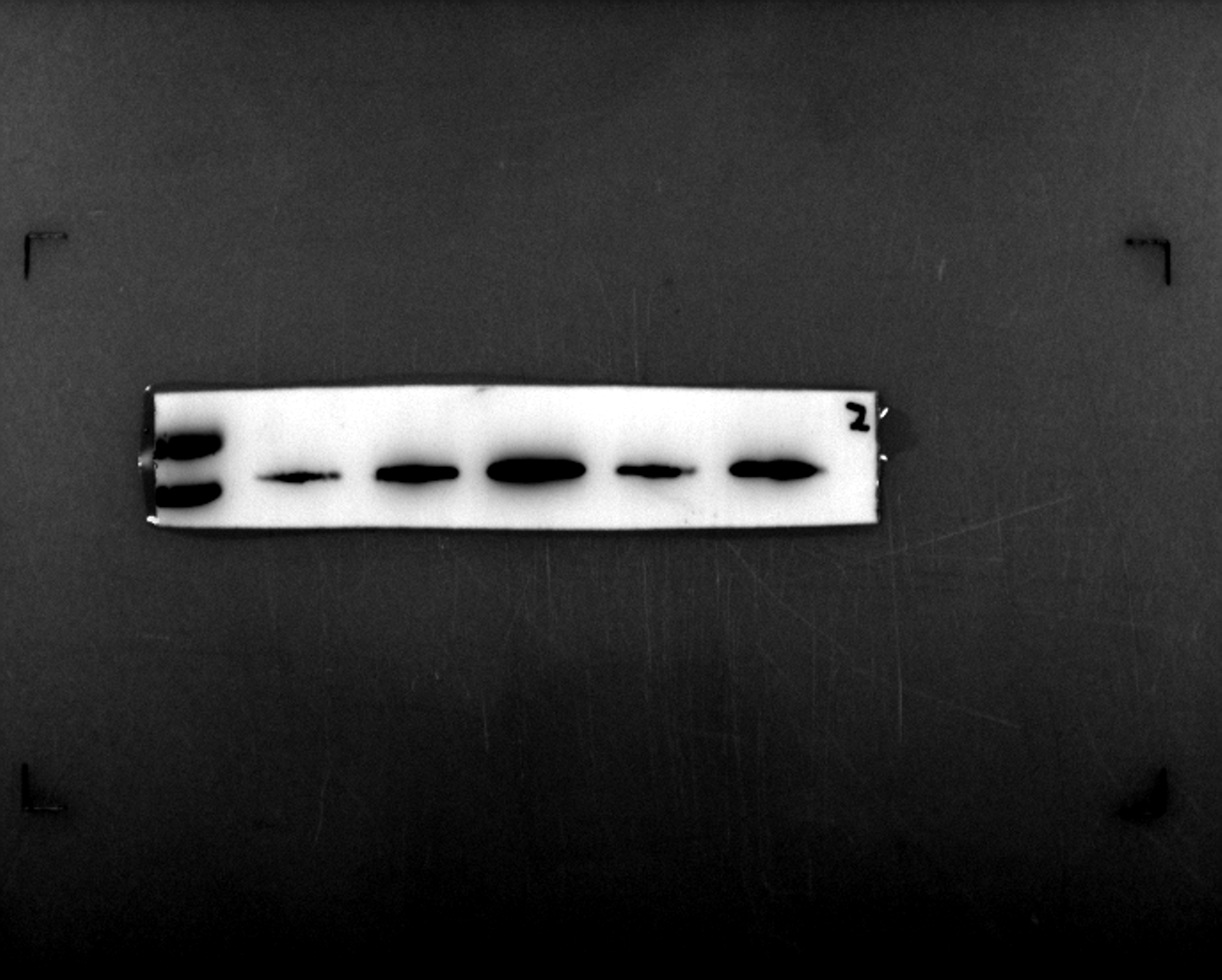


**40kDa**

**50kDa**

**43kDa**

**Supplementary Figure 5.** The additionally original picture of **Figure 4D** (*Rage* Protein in osteoblasts were detected by western blot analysis).


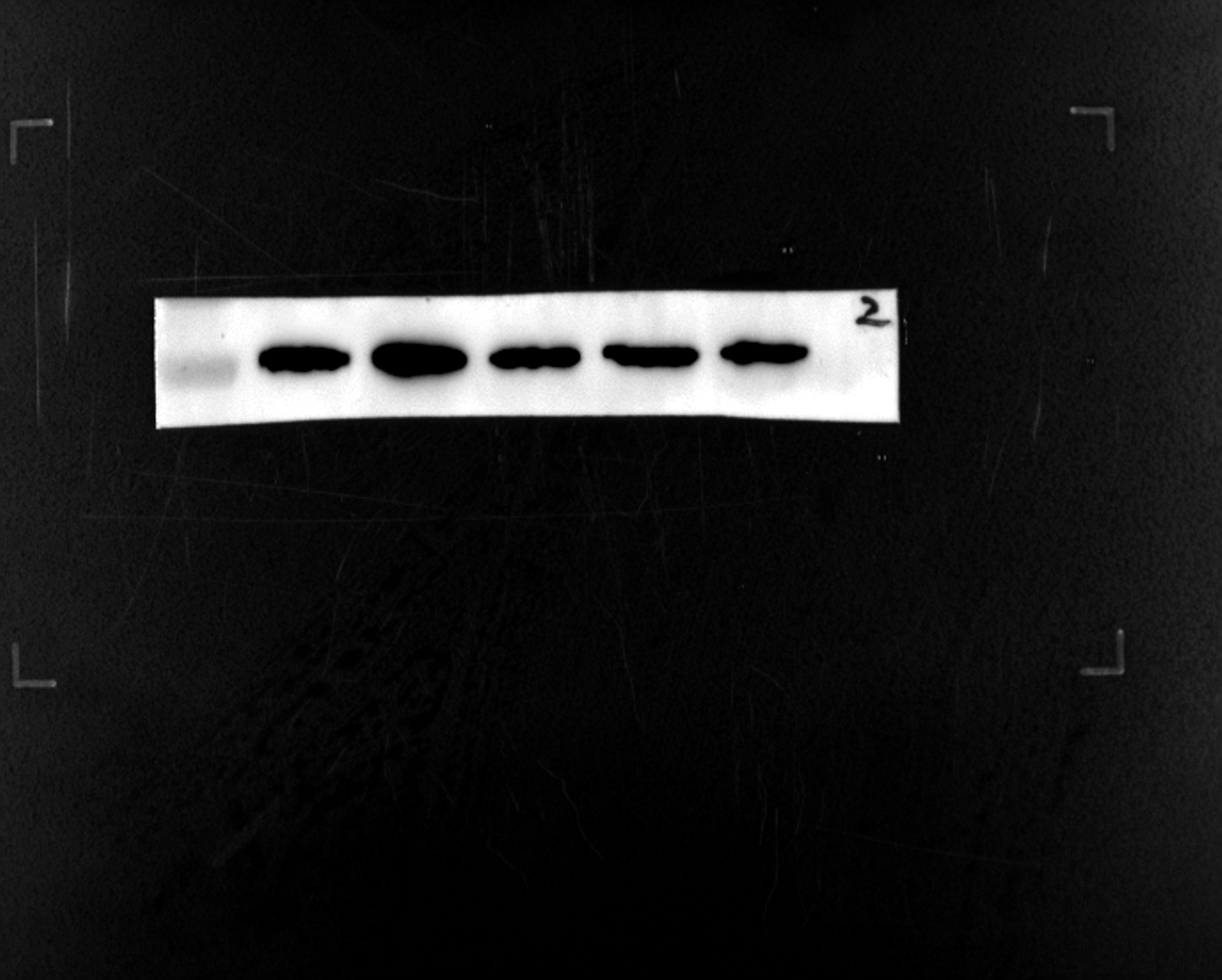


**35kDa**

**25kDa**

**23kDa**

**Supplementary Figure 6.** The additionally original picture of **Figure 4D** (*Cyclophilin B* Protein in osteoblasts were detected by western blot analysis).


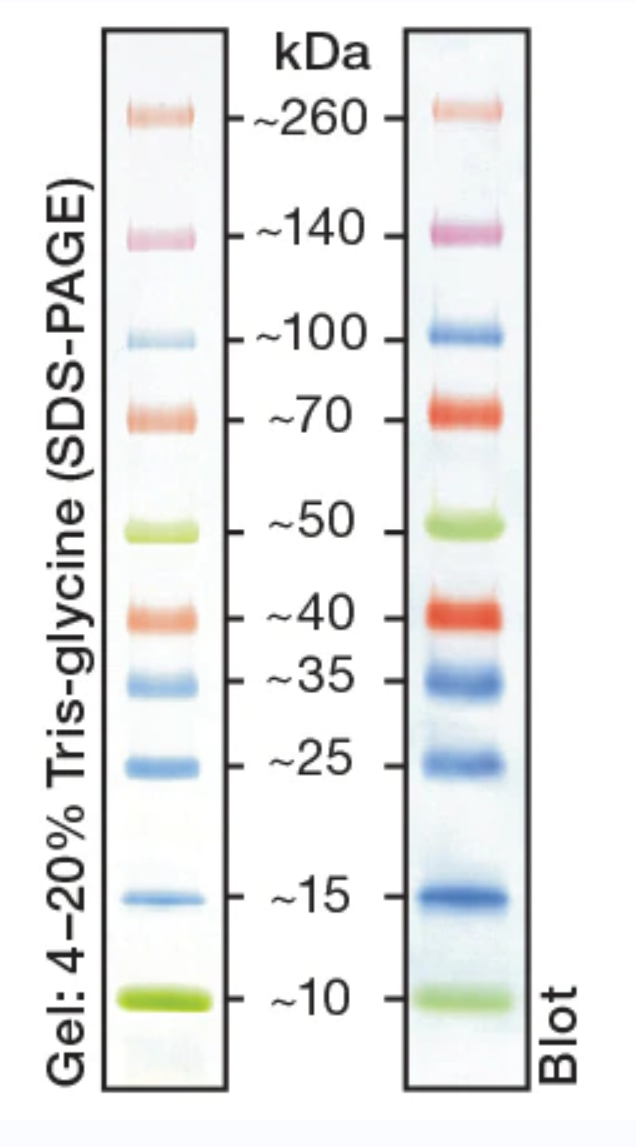


**Supplementary Figure 7.** The protein marker (ThermoFisher, 26634).
